# From polycystic ovary syndrome to polyendocrine metabolic ovarian syndrome: rationale, evidence, and implications of a global name change

**DOI:** 10.3389/fgwh.2026.1881840

**Published:** 2026-08-17

**Authors:** Abdinasir Adam Shidane, Zakariye Isak Mohamed, Muhammad Umair Manzoor, Muhammad Owais, Sakina Ali

**Affiliations:** 1 Faculty of Medicine and Surgery, Somali National University, Mogadishu, Somalia; 2 Department of Medicine, Wah Medical College, National University of Medical Sciences, Rawalpindi, Pakistan

**Keywords:** diagnosis, hyperandrogenism, nomenclature, PCOS, PMOS, polyendocrine metabolic ovarian syndrome, stigma, women's health

## 1 Introduction

Polycystic ovary syndrome (PCOS) affects approximately 9%–13% of women of reproductive age worldwide, an estimated 170 million individuals, depending on which diagnostic criteria are applied (1, 2). It is one of the most common endocrine disorders in this population. Even though it is so common, PCOS has often been underdiagnosed, care has been fragmented, and research funding has been limited (3, 4). Part of the problem comes from its name, which can be misleading (3, 4). The term “polycystic ovary” suggests that the disorder involves abnormal ovarian cysts, but this is not actually the case. Instead, ultrasound usually shows arrested follicular development caused by neuroendocrine and metabolic problems, which is a different issue (5).

**Figure 1:**
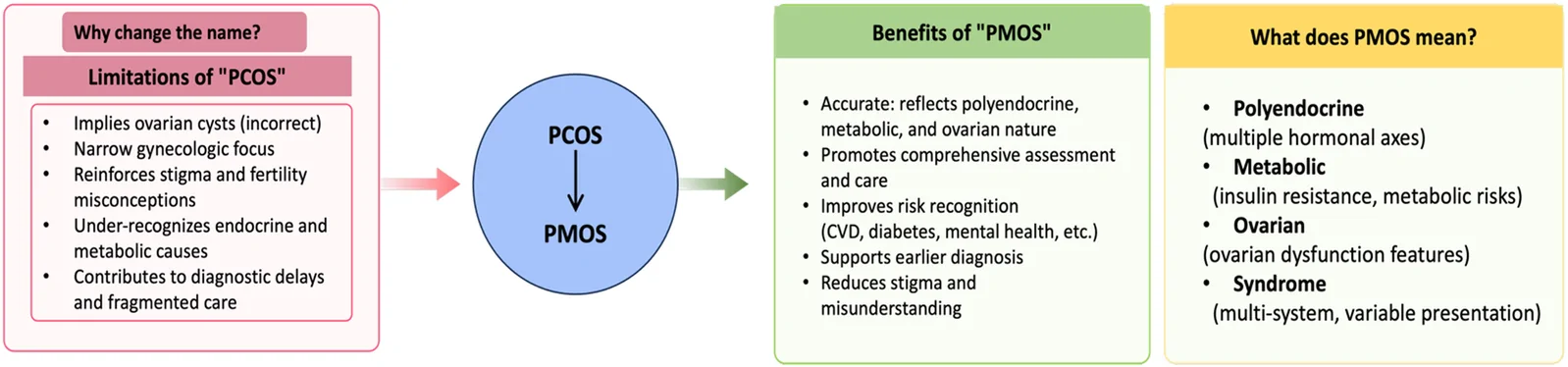
Rationale for the transition from polycystic ovary syndrome (PCOS) to polyendocrine metabolic ovarian syndrome (PMOS). The figure summarizes the key limitations of the term PCOS, the scientific and clinical rationale supporting PMOS, and the broader endocrine, metabolic, and ovarian dimensions reflected in the proposed nomenclature.

In May 2026, Teede et al. published the outcome of an unprecedented global consensus process involving 56 organisations, 14,360 survey responses from patients and health professionals across all world regions, modified Delphi surveys, nominal group workshops, and marketing analysis (5). The resulting new name, polyendocrine metabolic ovarian syndrome (PMOS), omits the misleading cyst reference and explicitly captures the condition's endocrine, metabolic, and ovarian dimensions. This opinion article evaluates the evidence base underpinning this rename, examines its clinical and psychosocial implications, and identifies the implementation priorities essential to converting a nomenclature change into genuine improvement in patient outcomes (Figure 1).

## 2 Recognising the polyendocrine and metabolic reality

The historical framing of PCOS as a primarily gynecological or ovarian disorder has led to its broader effects being treated as separate issues. However, international guidelines have tried to address this. The 2018 and 2023 International Evidence-Based Guidelines recognize a range of metabolic, reproductive, psychological, and skin-related features and note that gaps in practice and delays in diagnosis persist even with these guidelines (3, 4). The 2023 update added anti-Müllerian hormone (AMH) as another option for adult diagnosis, highlighted the importance of metabolic and cardiovascular risks, and placed greater emphasis on psychological health (4). Yet clinicians continue to show variable awareness of diagnostic criteria and the breadth of PMOS features (6).

PMOS involves multiple interacting endocrine axes. Elevated gonadotropin-releasing hormone pulse frequency drives excess luteinizing hormone secretion, augmenting ovarian androgen production. Insulin resistance is present in approximately 85% of affected individuals, including 75% of lean women, and acts synergistically to amplify androgen excess and disrupt steroid hormone balance (5, 7, 8). Abnormal AMH signaling, altered adipokine profiles, and excess adrenal androgens further compound endocrine dysregulation (5, 7). Cardiometabolic sequelae are well-documented: type 2 diabetes, dyslipidemia, metabolic dysfunction-associated fatty liver disease (MAFLD), hypertension, and elevated cardiovascular disease risk represent the downstream morbidity that the old name systematically obscured (3, 4, 7). Calling the condition “polyendocrine” is based on evidence, not just style. Recently, experts have suggested shifting from glucose-focused to insulin-focused screening for PMOS. This change could help identify metabolic and reproductive problems earlier, which the old name did not highlight (8). Including “metabolic” in the name makes it clear that checking insulin sensitivity, blood sugar, and cardiovascular risk should be a main part of diagnosis, not an afterthought.

## 3 Stigma, psychological burden, and the case for accurate language

There is strong evidence supporting the psychosocial reasons for renaming PCOS, beyond just scientific accuracy. Qualitative studies using social constructionism have shown that women with PCOS often face serious biopsychosocial challenges, feel socially isolated, and are frequently dissatisfied with their healthcare experiences (9). Studies in clinical settings have consistently found higher rates of depression, anxiety, and stress among people with PCOS, with the odds of anxiety and depression much higher than in those without the condition, regardless of cultural background (10, 11). A systematic review also showed that all major meta-analyses agree on the increased psychological burden in PCOS, such as body image issues, eating disorders, and psychosexual problems. Despite this, many clinicians are still not well informed about these links (12).

The term “polycystic ovary” has added to the challenges faced by patients, especially in cultures where fertility is closely tied to social identity and self-worth (5). By removing the cyst reference and focusing on endocrine and metabolic aspects, PMOS shifts attention away from reproductive failure as the main feature of the condition. Still, it is important to communicate carefully: patient education materials should avoid linking “metabolic” to weight-blame, since the metabolic features of PMOS are substantially genetically driven and not attributable to lifestyle alone (3, 5).

## 4 Diagnostic delays: A structural problem the name change must address

Up to 70% of people with PMOS remain undiagnosed globally (5, 6). Several factors contribute to this, including confusion among clinicians about which criteria to use in different situations (6), differences in diagnostic cut-offs across the NIH, Rotterdam, and AE-PCOS criteria (2), limited access to labs in some areas, and poor communication between patients and clinicians, partly because of the condition's name. A qualitative study in Australia found that clinicians varied widely in how they diagnosed and explained PCOS, and many were unsure about the risks of both overdiagnosis and underdiagnosis (6). Even where international guidelines are well known, there are still gaps between evidence and practice (3, 4).

Adopting PMOS should help make diagnoses clearer by shifting the focus from just ovarian morphology to the patient's metabolic and endocrine profile. Still, simply changing the name is not enough to solve the underlying issues. There needs to be real investment in educating clinicians in both primary care and specialist roles, providing in point-of-care AMH testing for settings without ultrasound access, creating patient information that fits different cultures, and improving diagnostic pathways, particularly in low- and middle-income countries (LMICs), where involvement in the naming consensus process was recognized as limited (5).

## 5 Discussion

Changing the name from PCOS to PMOS comes from scientific research and international teamwork. The new name gives a clearer picture of the condition, which includes several hormone imbalances, metabolic changes, and ovarian symptoms. This update helps fix a misleading name that has confused in diagnosis and research, and reinforced stigma for over seventy years.

The consensus methodology employed by Teede et al. (5) merits specific recognition as a model for the field. The structural integration of patient voices not as token consultation but as co-governance through organisations such as Verity, combined with iterative Delphi surveys, nominal group technique, and marketing analysis, generated a process whose legitimacy rests on transparency and representativeness. This stands in contrast to previous renaming attempts that failed to achieve traction through lack of inclusive global leadership and the absence of a coordinated implementation strategy (5). Future nomenclature reforms in other conditions should study and adapt this model.

There are several important challenges to consider as implementation moves forward. First, the implementation strategy covers eight stages, including ICD coding, aligning electronic health records, and updating the International Guidelines for 2028, but it primarily applies to high-income countries (5). In low- and middle-income countries, where diagnostic resources are limited, and the condition is becoming more common, there is a need for separate, co-designed workstreams with dedicated support. The global research agenda for PMOS should focus on equity-conscious research, including collecting normative data and validating diagnostic thresholds in South and East Asian, African, and Latin American populations.

Second, the term “polyendocrine” is understood differently in various languages and cultures. The authors recognize the importance of careful translation (5). However, prospective qualitative studies to assess how people understand translated names before using them widely would help ensure accurate implementation. Communities that speak Arabic, Hindi, Amharic, Portuguese, and Swahili, among others, are major patient groups who need communication that fits their cultures.

Third, the three-year transition period should incorporate pre-specified evaluation milestones: Does time-to-diagnosis change? Does patient-reported understanding of the condition improve? Do research funding allocations shift toward metabolic and endocrine features? These outcomes, measured prospectively, will determine whether PMOS constitutes genuine progress or a renaming without transformation.

### 5.1 Potential drawbacks and controversies

While the scientific and psychosocial rationale for PMOS is well-founded, intellectual honesty requires engagement with legitimate concerns. First, the three-year transition period during which both PCOS and PMOS will be in simultaneous clinical and coding use creates real risks of diagnostic fragmentation, inconsistent insurance coding, and clinician confusion, costs that previous disease renames have also incurred and that require active mitigation. Second, patient advocacy groups have raised concerns that “polyendocrine” may be less immediately accessible than “polycystic ovary” for patients with lower health literacy, particularly before multilingual communication materials are validated. Third, the eight-stage implementation roadmap is weighted toward infrastructure, ICD coding, electronic health records, and specialist guideline updates, which are more relevant to high-income settings, raising equity concerns for the LMICs where PMOS prevalence is rising most rapidly. Fourth, incorporating “metabolic” into the name carries the risk of inadvertently reinforcing weight-blame narratives, since metabolic dysfunction is frequently conflated in lay discourse with lifestyle choices, despite the condition's substantially genetic architecture (3, 5, 7). Patient communication materials must explicitly pre-empt this interpretation.

Finally, the evidence supporting PMOS management is still mostly low to moderate in quality (3, 4). The name change creates an opportunity, indeed an obligation, to align future research design with the polyendocrine framing: multi-axis mechanistic studies, phenotype-stratified intervention trials, and longitudinal cohort studies tracking outcomes across the full lifespan and across diverse populations should be prioritised. The International PCOS Guideline clinical research roadmap, developed with input from both patients and health professionals, provides a strong framework for this agenda (13).

A critical epistemic limitation of the current article and of renaming advocacy more broadly is the absence of prospective outcome data specific to PMOS. The evidence base for whether renaming translates into measurable reductions in diagnostic delay, improvements in patient-reported outcomes, or greater treatment adherence will only be generated during the three-year transition period and beyond. Historical analogues are instructive but not directly transferable: the reclassification of manic-depressive illness to bipolar disorder in 1980 was associated with reduced perceived stigma and improved treatment-seeking over time; the renaming of non-insulin-dependent diabetes mellitus to type 2 diabetes mellitus aligned clinical discourse with insulin-based screening paradigms; and the WHO's 2013 classification of obesity as a chronic disease shifted clinical management toward medical rather than volitional frameworks. However, in each case, the rename was a necessary but not sufficient condition; structural investment in education, guideline reform, and patient communication drove the realized benefit. The present manuscript, therefore, avoids claiming that PMOS will improve outcomes; rather, it argues that PMOS creates the enabling nomenclature conditions for such improvement, contingent on rigorous implementation and prospective evaluation.

## 6 Conclusion

Polyendocrine metabolic ovarian syndrome is a more accurate name for this condition, which is a polyendocrine disorder with widespread metabolic effects and ovarian symptoms. It affects 170 million women worldwide. The global consensus that led to this name sets a new standard for evidence-based, patient-focused naming. To make the most of this change, ongoing efforts are needed in clinician education, fair implementation, clear communication in many languages, and careful tracking of its impact. PMOS is not just a better name; it also supports the comprehensive, multidisciplinary care that people with this condition have needed for a long time.

## References

[1] Salari N Nankali A Ghanbari A Jafarpour S Ghasemi H Dokaneheifard S. Global prevalence of polycystic ovary syndrome in women worldwide: a comprehensive systematic review and meta-analysis. Arch Gynecol Obstet. (2024) 310:1303–14. 10.1007/S00404-024-07607-X 38922413

[2] Skiba MA Islam RM Bell RJ Davis SR. Understanding variation in prevalence estimates of polycystic ovary syndrome: a systematic review and meta-analysis. Hum Reprod Update. (2018) 24:694–709. 10.1093/HUMUPD/DMY022 30059968

[3] Teede HJ Misso ML Costello MF Dokras A Laven J Moran L. Recommendations from the international evidence-based guideline for the assessment and management of polycystic ovary syndrome. Hum Reprod. (2018) 33:1602–18. 10.1093/HUMREP/DEY256 30052961 PMC6112576

[4] Teede HJ Tay CT Laven JJE Dokras A Moran LJ Piltonen TT. Recommendations from the 2023 international evidence-based guideline for the assessment and management of polycystic ovary syndrome. J Clin Endocrinol Metab. (2023) 108:2447–69. 10.1210/CLINEM/DGAD463 37580314 PMC10505534

[5] Teede HJ Khomami MB Morman R Laven JSE Joham AE Costello MF. Polyendocrine metabolic ovarian syndrome, the new name for polycystic ovary syndrome: a multistep global consensus process. Lancet. (2026) 407:2329–39. 10.1016/S0140-6736(26)00717-8 42119588

[6] Copp T Muscat DM Hersch J McCaffery KJ Doust J Mol BW. Clinicians’ perspectives on diagnosing polycystic ovary syndrome in Australia: a qualitative study. Hum Reprod. (2020) 35:660–8. 10.1093/HUMREP/DEAA005 32101283

[7] Stener-Victorin E Teede H Norman RJ Legro R Goodarzi MO Dokras A. Polycystic ovary syndrome. Nat Rev Dis Primers. (2024) 10:27. 10.1038/S41572-024-00511-3 38637590

[8] Parker J Briden L Gersh FL. Recognizing the role of insulin resistance in polycystic ovary syndrome: a paradigm shift from a glucose-centric approach to an insulin-centric model. J Clin Med. (2025) 14:4021. 10.3390/JCM14124021 40565766 PMC12194245

[9] Wright PJ Dawson RM Corbett CF. Social construction of biopsychosocial and medical experiences of women with polycystic ovary syndrome. J Adv Nurs. (2020) 76:1728–36. 10.1111/JAN.14371 32215949

[10] Asdaq SMB Yasmin F. Risk of psychological burden in polycystic ovary syndrome: a case control study in Riyadh, Saudi Arabia. J Affect Disord. (2020) 274:205–9. 10.1016/J.JAD.2020.05.086 32469805

[11] Tabassum F Jyoti C Sinha HH Dhar K Akhtar MS. Impact of polycystic ovary syndrome on quality of life of women in correlation to age, basal metabolic index, education and marriage. PLoS One. (2021) 16:e0247486. 10.1371/JOURNAL.PONE.0247486 33690645 PMC7946178

[12] Alur-Gupta S Dokras A Cooney LG. Management of polycystic ovary syndrome must include assessment and treatment of mental health symptoms. Fertil Steril. (2024) 121:384–99. 10.1016/j.fertnstert.2024.01.018 38244713

[13] Teede HJ Gibson M Laven J Dokras A Moran LJ Piltonin T. International PCOS guideline clinical research priorities roadmap: a co-designed approach aligned with end-user priorities in a neglected women’s health condition. eClinicalMed. (2024) 78:102927. 10.1016/J.ECLINM.2024.102927 PMC11614868 39634033

